# An Improved STC-Based Full Coverage Path Planning Algorithm for Cleaning Tasks in Large-Scale Unstructured Social Environments

**DOI:** 10.3390/s24247885

**Published:** 2024-12-10

**Authors:** Chao Wang, Wei Dong, Renjie Li, Hui Dong, Huajian Liu, Yongzhuo Gao

**Affiliations:** State Key Laboratory of Robotics and System, Harbin Institute of Technology (HIT), Harbin 150001, China; 19b908039@stu.hit.edu.cn (C.W.); dongwei@hit.edu.cn (W.D.); 22b908020@stu.hit.edu.cn (R.L.); dongh@hit.edu.cn (H.D.); hjliu@stu.hit.edu.cn (H.L.)

**Keywords:** social environments, coverage path planning, STC, back tracking, multi-robot

## Abstract

Some large social environments are expected to use Covered Path Planning (CPP) methods to handle daily tasks such as cleaning and disinfection. These environments are usually large in scale, chaotic in structure, and contain many obstacles. The proposed method is based on the improved SCAN-STC (Spanning Tree Coverage) method and significantly reduces the solution time by optimizing the backtracking module of the algorithm. The proposed method innovatively introduces the concept of optimal backtracking points to sacrifice the spatial complexity of the algorithm to reduce its computational complexity. The necessity of backtracking in such environments is proved to illustrate the generalization ability of the method. Finally, based on secondary coding, the STC solution is explicitly expressed as a continuous and cuttable global path, which can be generalized to Multi-robot Covered Path Planning (MCPP) to avoid the path conflict problem in the multi-robot system, and the paths assigned to each robot have good balance. The method of this study is proven to be effective through simulations in various random environments and a real environment example. Compared with the advanced methods, the computational time is reduced by 82.47%.

## 1. Introduction

With the increasing severity of virus outbreaks in recent years [1], the demand for cleaning and disinfection work focused on isolating personnel in public spaces has emerged. The development of artificial intelligence technology and the widespread deployment of various robotic products have gradually increased people’s acceptance of using robots to handle tasks such as cleaning and disinfection [2,3,4]. As a result, many social environments have started utilizing robots for routine cleaning and disinfection tasks [5].

System operation efficiency and robustness are significantly improved using multi-robot coverage path planning (MCPP). Problems studied in this area are required to generate a path covering every point within the work area while avoiding obstacles. The common goals of the MCPP method recognized by most scholars are to minimize the total path length, reduce the secondary coverage rate, minimize the number of turns, and reduce the calculation time. The optimization problem of MCPP is a combinatorial optimization problem with high computational complexity, and is considered NP-hard [6]. As the map scale increases and the percentage of obstacles increases, it becomes significantly more challenging. Therefore, it is important to find a feasible MCPP solution with lower computational complexity, reduced secondary coverage, and reduced turns [7,8].

This study focuses on large unstructured social environments, such as luxury hotels and amusement parks [9], where the challenges of cleaning tasks are mainly reflected in three aspects: (1) these environments are unstructured, large in scale, and contain many obstacles. Due to their large scale, it is difficult for a single robot to complete the task within a limited time. The large number of obstacles causes traditional algorithms to generate many sub-areas in the segmentation stage. The scheduling between these sub-areas becomes a Traveling Salesman Problem (TSP), resulting in high computational complexity [10,11]. It is difficult for the Boustrophedon (BP) method to adapt to environments with many obstacles, resulting in a large amount of secondary coverage and an increase in the total path length [12]. (2) These environments often contain many pedestrians and are human–robot coexistence environments. This makes high-complexity computational methods unable to handle the problem of path conflicts with humans due to their insufficient re-planning capabilities [13]. Therefore, the MCPP method based on mixed integers is also not applicable to the environment. (3) Cleaning robots used in industry experience material wear during the cleaning process, such as excessive use of cleaning fluid or brush wear [14]. Therefore, the invalid paths caused by secondary coverage should be reduced as much as possible. Furthermore, their mobile platforms have nonholonomic constraints, meaning they cannot perform lateral movements [15], thus reducing the number of turns; secondary coverage is crucial to minimizing the length of invalid paths.

In order to address these challenges and improve the efficiency of coverage path planning (CPP) operations, and to ensure that our method can be extended to multi-robot systems, a method to reduce the computational complexity of STC by optimizing the backtracking module is proposed. In Section 3.1, this study first constructs a mathematical model for solving CPP for a gridded environment to illustrate the input requirements of this method. In Section 3.2, a feasible framework is proposed to illustrate the relationship between the various modules of the proposed method. In Section 3.3, the concept of optimal backtracking points is innovatively introduced. By optimizing the STC backtracking module, the effect of sacrificing the algorithm space complexity to reduce the computational complexity is achieved. In Section 3.4, secondary encoding is used to make the STC solution explicit as a continuous coverage path, and then balanced cutting is performed. Finally, in Section 3.5, the technical solution for deploying the proposed algorithm to actual robots is introduced.

The contributions of this study are as follows:

(1) An improved STC algorithm is proposed, which reduces the computational complexity of the algorithm by optimizing the backtracking module. And by proving the necessity of backtracking, the generalization ability of the method to random environments is indirectly explained.

(2) A method is proposed to explicitly express the solution of STC as a continuous global reference path using secondary coding and then, distribute the path to the multi-robot system through balanced cutting. This method has been proven to obtain the optimal solution for balance.

## 2. Related Works

The requirements of the CPP problem vary in priority depending on the scenario. In some applications, such as perception-based coverage [16], 3D scanning [17] or other tasks that allow for repeated coverage of the same point, increase the total path length; however, the demand on the robot’s perception performance is significantly reduced. On the other hand, for another class of applications, such as agriculture, demining, and 3D printing, secondary coverage of the same point should be avoided to save energy or prevent damage. For example, in agricultural applications like ground leveling, passing over already processed areas could lead to excessive soil compaction [18]. These methods are more effective for small-sized areas or obstacle-free environments.

As a classic method for online CPP, the traditional randomly growing STC algorithm is a well-established approach to achieving complete coverage with low secondary coverage rates [19]. After a period of research, scholars have provided feasible solutions for both online and offline settings [20,21]. The advantage of the online spiral STC is that when there are no obstacles around the tree corresponding to the coverage area, new branches are directly generated. This method avoids redundant scanning and reduces the solving time. Such online methods ensure a high path update frequency, allowing the system to avoid dynamic obstacles in the environment. For single-robot system coverage path planning, these methods offer significant advantages in terms of system robustness. Some of the more advanced online path-planning methods are also capable of achieving complete coverage, particularly in narrow or unexplored environments [22,23]. However, they do not consider the potential path conflicts between multiple robots during area scheduling in multi-robot cooperative complete coverage. Some scholars have also used data-driven methods [24], physical model-based methods [25], and artificial intelligence methods to produce valuable results in solving local planning problems [26,27,28,29,30].

The sensitivity of the BP method to obstacles in the environment makes coverage path planning in such settings rely on area segmentation. As the number of obstacles or non-convex polygonal regions in the environment increases, the number of segmented areas also increases, indirectly turning the scheduling between areas into a TSP. In multi-robot systems, there is also the issue of path conflicts during scheduling. To address this, the system often maintains a priority queue for the robots, where the lower-priority robot stops and waits in case of a path conflict. However, this extends the total system operation time. There are also some offline coverage methods that allow for a higher secondary coverage rate. These methods focus on scenarios where the sensor range or the effective working area is large, making secondary coverage inevitable during the complete coverage process, such as signal coverage tasks performed by a group of robots following a moving object. The method based on regional segmentation has also been discussed by many scholars, but it also leads to the problem of scheduling robots between sub-regions [31,32].

For offline methods targeting multi-robot systems, a representative approach is the Mixed Integer-based MSTC [33,34,35]. This method is insensitive to the shape and distribution of obstacles in the environment and can handle weighted scenarios. However, its high computational complexity makes it unsuitable for large-scale environments.

## 3. Methodology

### 3.1. Problem Description

Suppose we are given a robot tasked with covering a finite environment using a CPP approach, where the environment’s free space is denoted as S, and the robot’s limited coverage footprint is denoted as the side length of the unit grid cell in the map used in this study. We also assume that the robot can determine its position within the environment and is equipped with sufficiently large distance sensors to detect whether neighboring cells are occupied. This allows the robot to avoid obstacles based on its local map when performing cleaning and disinfection tasks, thereby enhancing the system’s robustness against external disturbances. The parameters used in the study are illustrated in Table 1.

Based on the above assumptions, the problem under discussion is to find a solution that minimizes the total time consumed for the coverage task. To simplify the problem, this study does not consider the cases of uneven speed or system restarts due to crashes during the robot’s operation, meaning that the path length is equivalent to the time consumed for the task. To reduce conflicts in the coverage paths of different robots, the study assumes that during the robots’ collaborative work, the spatial intersection of any two robots in the system must not exceed a threshold $S_{t}$. The objective function and constraints of this study are as follows:

∀r∈R, ∀i∈R, ∀j∈R

minimize w(1)

s.t. w_r ≤ w (2)

(3) $$S_{1}\cup S_{2}\cup \cdots \cup S_{r}=S_{all}$$

(4) $$v_{r}\in S_{r}$$

(5) $$w_{r}=\sum v_{r}$$

(6) $$S_{r}\neq \emptyset$$

(7) $$S_{i}\cap S_{j}\leq S_{t}$$

We summarize the above formulas as follows:

Equation (1): Ensure that the solution to the MCPP problem is a near-optimal solution among feasible solutions.

Equation (2): Describes the operation time of the MCPP solution, which is the time taken by the robot with the longest operation time in the system.

Equations (3)–(5): Ensure that the total coverage area is the sum of the areas covered by each robot, while ignoring redundant paths due to secondary coverage when evaluating the total coverage.

Equations (6) and (7): Ensure that each robot can complete its assigned coverage task without path conflicts that could lead to higher-order deadlock in the multi-robot system, causing task failure.

**Figure 1:**
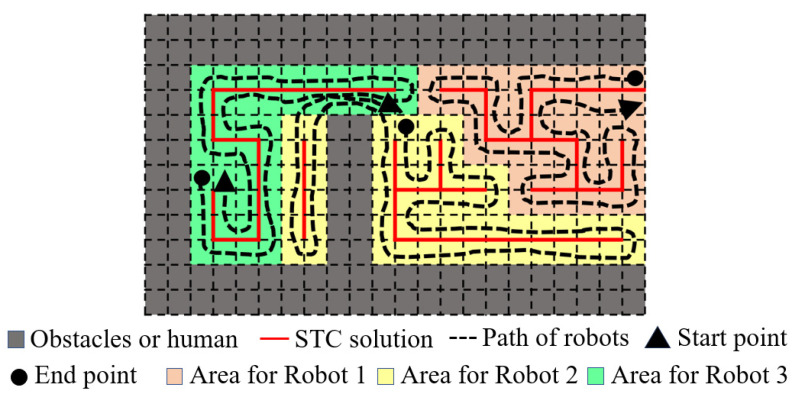
Schematic diagram of CPP.

In addition to the above constraints, we specify that the robots are ground differential-drive robots with complete kinematic constraints, meaning that the discretized path points can be interpolated to enable accurate tracking. The schematic diagram is shown in Figure 1.

### 3.2. Proposed Solution Framework

**Figure 2:**
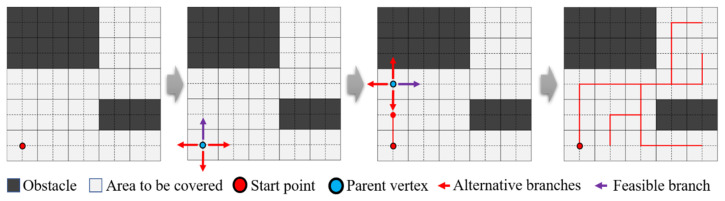
The basic principles of STC and a representation of terms used in this study.

To better introduce this method, the basic principles of STC and the terminology used in this study are briefly explained, as shown in Figure 2. In random scenarios where an STC solution is needed, the obstacle distribution and starting point are loaded. Then, the starting point is used as the parent vertex to search for alternative branches in the surrounding area. Feasible branches are determined through evaluation, and the process continues until the entire scene is fully covered.

**Figure 3:**
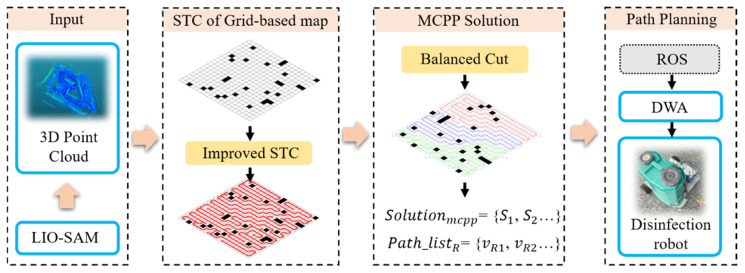
A feasibility framework based the proposed method.

The proposed solution is expected to improve solving speed, based on two key ideas: (1) Considering the secondary coverage of the solution at the first, there is no STC method without secondary coverage that can achieve complete coverage in any environment from any starting point without requiring backtracking. We support this with a simple proof, as shown in Proposition 1. The traditional backtracking mechanism involves traversing backward through already searched STC nodes, finding the nearest one to the current point that can generate a new branch, and continuing to expand from there until full coverage is achieved. To address this problem, we implemented a list of alternative backtracking points, allowing us to more quickly find feasible root vertices for new branches, thereby reducing computation time, as shown in Section 3.3. (2) According to Equation (2), the problem can be transformed into minimizing the working time of the robot with the longest operation time in a multi-robot system. A more balanced distribution of the area to be covered can significantly improve this metric, and reducing the rate of double coverage indirectly lowers the overall workload, which is also meaningful. The proposed method introduces a technique for balanced path segmentation after generating explicit coverage paths based on the STC. This method has been proven to achieve a maximum gap of $G_{opt}$ compared to the optimal solution, as shown in Proposition 2. To account for its deployment in practical applications, a feasibility framework based on the proposed method is proposed, as illustrated in Figure 3, in which the yellow parts are our contributions.

**Proposition** **1.** *There is no STC method that can completely cover any environment with any starting point and satisfy the following two constraints. Constraint 1: No backtracking occurs. Constraint 2: No secondary coverage.*

**Figure 4:**
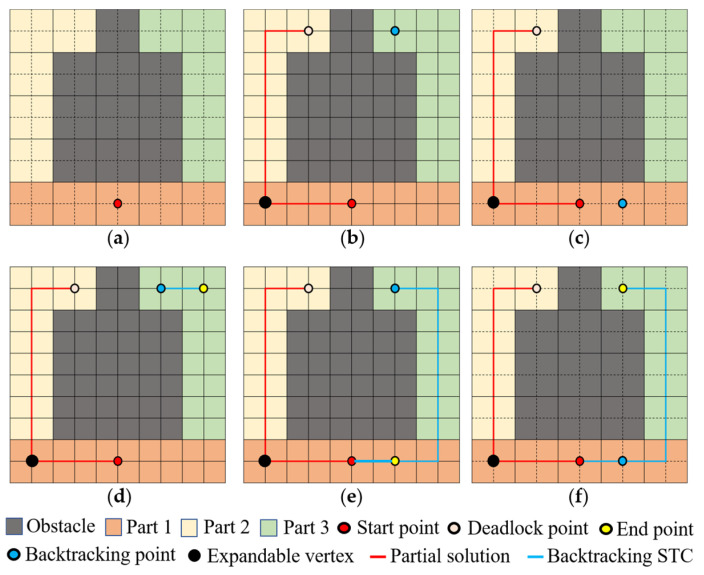
Illustrative diagram of a case with an inevitable backtracking situation: (**a**) illustrative diagram of a scenario used as a counterexample; (**b**) illustrative diagram of a deadlock situation; (**c**) illustrative diagram of an alternative backtracking method for a deadlock situation; (**d**) a solution method for the generated tree after backtracking; (**e**) an example of complete coverage after backtracking; (**f**) another example of complete coverage after backtracking.

**Proof of Proposition** **1.** Assume that there is an STC method that can achieve full coverage from any starting point in any environment and satisfy the above two conditions. To refute this hypothesis, we can find a counterexample, as shown in Figure 4. First, consider the case where quadratic coverage does not occur. After the first part is covered, no matter which of the two available directions in the figure is chosen to expand the tree, a deadlock area will inevitably be formed. If you cover part 1 first and then expand the tree to part 2, a deadlock will occur after covering part 2, and backtracking will be necessary to cover part 3. Considering the case where full coverage is not achieved by backtracking, still referring to the counterexample, based on the STC solution process, it is necessary to re-pass the generated branches, then secondary coverage will occur, which contradicts the hypothesis. Therefore, the hypothesis is not established, and the proposition is proved. □

The proposed method, when the number of obstacles in the environment, as well as the length and width of the environment’s boundaries, are known, can demonstrate the difference in coverage between its solution and the optimal solution in a grid map.

**Proposition** **2.** *For a grid map where the entire coverage area is connected, the solution obtained by the proposed method can have a maximum difference from the optimal solution, denoted as $G_{opt}$ which satisfies Equation (8).*

(8) $$G_{opt}=3N_{obs}+3\left(l_{evn}+h_{evn}-4\right)/2$$

**Figure 5:**
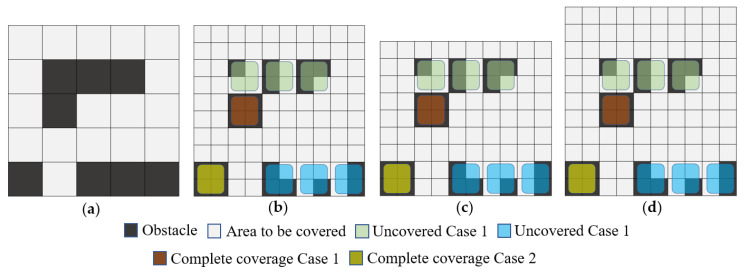
Schematic diagram of the principles behind the occurrence of incomplete coverage: (**a**) an example scenario; (**b**) example of situations leading to incomplete coverage when restoring STC grid maps to original grids; (**c**) a situation where an odd edge size prevents complete coverage by STC; (**d**) another situation where an odd edge size prevents complete coverage by STC.

**Proof of Proposition** **2.** When the input scene is any form of a grid-based map $C_{1}$, let its length and width be $l_{evn}$ and $h_{evn}$, respectively. It can be transformed into the input required for the STC algorithm. The implementation method is to complete $C_{1}$ into a square shape and expand the obstacle regions, converting the original map into a grid map $C_{2}$ with both $l_{evn}$ and $h_{evn}$ being even, as shown in Figure 4a. Then, a trivial solution can be obtained using traditional STC method [19]. Thus, complete coverage of $C_{2}$ has been achieved. The areas that have not achieved 100% coverage can only exist in six possible scenarios near obstacles or boundaries, as shown in Figure 5a,b. Additionally, there are uncovered regions at the boundaries caused by the parity of the scene dimensions, as illustrated in Figure 5c,d. If the number of occupied grid cells by obstacles is $N_{obs}$, then the corresponding uncovered area in $C_{1}$ does not exceed 3$N_{obs}$ cells. In the worst-case scenario, as all the obstacles are not adjacent to the boundary, the total uncovered area can be represented as 3$N_{obs}$. Similarly, for the boundaries, the worst-case scenario can be calculated as Equation (8). Additionally, complete coverage condition 1 indicates that the obstacle occupancy in the original grid map is consistent with that in the grid map used for STC solving, while complete coverage condition 2 means that the redundancy of obstacles in the four corners should be considered during calculations. The proposition is proven. □

### 3.3. Improved STC Based on Optimized Backtracking Module

The STC method has many variations. To reduce the number of turns [36], we improve upon the SCAN-STC method as a reference [37]. The underlying logic of this type of algorithm is similar to traditional methods, continuously updating the parent vertex to find the next branch vertex and improving its random search into a scanning search. This approach reduces the number of turns while ensuring a low rate of double coverage. When the branches of the spanning tree cannot be extended, resulting in a deadlock, a backtrack is performed. This vertex is referred to as a deadlock point in this study, and the partially completed STC solution is termed the STC intermediate solution.

The backtracking method involves maintaining a vertex list during the expansion of the spanning tree. When backtracking is necessary, it scans the current tree’s vertices for extensibility in reverse order along the list. When an extendable vertex is found, it is used as the new root vertex for the branch, thus regenerating the tree. This point is called a feasible backtracking point in this study. This point needs to have two properties. The first is that the point must be in the already generated tree, and the second is that a new branch can be generated with this point as the new root point and it does not overlap with the already generated tree.

**Figure 6:**
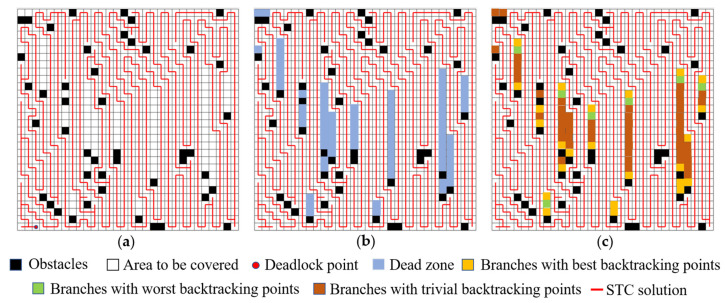
The principle of STC backtracking: (**a**) illustrative diagram of the situation after a deadlock occurs; (**b**) areas that require backtracking for coverage; (**c**) schematic diagram of the principles behind different backtracking methods leading to varying backtracking counts.

This method not only avoids the issue of the traditional STC method for obtaining optimal solutions, where each expansion requires searching in all four directions, transforming the overall problem into an NP-hard problem, but also retains the advantages of high total coverage and low secondary coverage of the STC, as well as the proximity of the starting and ending points. In a coverage area of the same size, the original backtracking method often encounters backtrack points located near the starting point. When a deadlock occurs, it requires traversing a longer backtrack sub-list to continue expansion. The principle is illustrated in Figure 6. A pattern was observed in the analysis of multiple different STC solutions in a random scene: if a feasible backtracking point can be obtained in a certain way that shortens the number of backtracks, this study refers to it as a superior backtracking point.

This type of backtracking point can be obtained using the following method: if a point is adjacent to an obstacle or boundary and belongs to an uncovered area, it can be connected to the intermediate solution in the reverse direction of the scanning path. The vertex on the connected intermediate solution becomes the superior backtracking point. As illustrated in Figure 6c, the corresponding uncovered points are referred to as backtracking reference points. In contrast, backtracking through non-superior backtracking points can easily lead to additional backtrack counts, as shown in Figure 6a,b.

The proposed method optimizes the backtracking module, with the overall idea of increasing spatial complexity and reducing computational complexity. Two additional new lists are maintained in addition to the original algorithm: the obstacle-adjacent point list and the superior backtracking list. The obstacle-adjacent point list is a static list that sequentially adds grid points to be covered around obstacles based on the SCAN-STC scanning direction. The superior backtracking list is derived from the obstacle-adjacent point list and also includes the original backtracking list, which is updated in real time. The update method involves removing duplicate points from the obstacle-adjacent point list during the process of the spanning tree continuously updating the intermediate solution.

When a deadlock occurs, the method no longer backtracks point-by-point along the STC intermediate solution. Instead, it directly takes the feasible backtracking point with the smallest index from the latest superior backtracking list as the vertex to initiate the generation of a new branch in the tree. This point is then removed from the superior backtracking list until the backtracking point list is empty, at which point the cycle ends. The pseudocode for this process is shown in Algorithm 1, and it works as follows.

The algorithm input includes the scene map and any starting point that is not within an obstacle, and it uses three lists: $P_{STC}$, $P_{opt\text{-}list}$, $P_{boundary\text{-}next}$ and $M_{c}$. $P_{STC}$ is the real-time updated STC intermediate solution list, confirmed in lines 18–50. $P_{opt\text{-}list}$ is the real-time updated superior backtracking point-list. $P_{boundary\text{-}next}$ is the obstacle-adjacent point-list. $M_{c}$ is used to store the cells around the real-time parent vertex during the STC solving process, with the labels $V_{L}$, $V_{U}$, $V_{R}$, and $V_{D}$ representing the cells to the left, top, right, and bottom of the parent vertex, respectively.

At the initial stage of the algorithm, a grid map $M_{R}$ is initialized to represent the set of space points $S_{r}$ to be covered, and the occupancy status of each cell is stored and labeled as UNOCCUPIED or OCCUPIED. The STC intermediate solution list starts empty, and as the algorithm iterates, it eventually covers all points in $S_{r}$, ultimately outputting the STC solution $P_{STC}$.

**Algorithm 1** Improved SCAN-STC to Cover the Area**Input:** Grid-based map $M_{R}$ **Output:** $\mathrm{STC}\;\mathrm{solution}:\;P_{STC}$$S_{r}\leftarrow M_{R}$(UNOCCUPIED);$M_{c}\leftarrow \left\{V_{L},\;V_{U},\;V_{R},\;V_{D}\right\}$; //Four points surrounding the current point$P_{opt\text{-}list}\leftarrow \left\{\;\right\}$; //Initialize the superior backtracking list.1:$P_{opt\text{-}list}\leftarrow \sum P_{boundary\text{-}next}$; //Load boundary points into the backtracking list.2:**For** $i=1:n^{2}$3:
$\mathrm{Iterate}\;\mathrm{through}\;\mathrm{all}\;\mathrm{points}\;\mathrm{in}\;\mathrm{the}\;\mathrm{scene},\;\mathrm{set}\;\mathrm{current}\;\mathrm{point}\;\mathrm{as}\;v_{c}$; 4:
**If** $v_{c}$ is an obstacle or boundary5:

$\mathrm{Set}\;\mathrm{the}\;\mathrm{four}\;\mathrm{surrounding}\;\mathrm{points}\;\mathrm{of}\;v_{c}$$\;\mathrm{as}\;M_{c}$;6:

**For** $v_{next}$$\leftarrow M_{c}$7:


**If** $v_{next}\notin P_{opt\text{-}list}$8:



**If** $v_{next}$ has not exceeded the boundary9:




Add the point to the end of the superior backtracking list; 10:




**End**
11:



**End**
12:


**End**
13:

**End**
14:
**End**
15:$\mathrm{Initialize}\;\mathrm{the}\;\mathrm{total}\;\mathrm{number}\;\mathrm{of}\;\mathrm{grid}\;\mathrm{cells}\;\mathrm{in}\;\mathrm{the}\;S_{r}$ to be covered as *h*;16:$\mathrm{Initialize}\;\mathrm{the}\;\mathrm{STC}\;\mathrm{iteration}\;\mathrm{threshold}\;I_{h}=h$;17:$\mathrm{Update}\;P_{opt\text{-}list}$;18:$\mathrm{Initialize}\;\mathrm{the}\;\mathrm{STC}\;\mathrm{starting}\;\mathrm{point}\;v_{i}$;19:$\mathrm{Initialize}\;\mathrm{the}\;\mathrm{STC}\;\mathrm{intermediate}\;\mathrm{solution}\;\mathrm{list}\;P_{STC}$;20:**While** i←h21:
$V_{c}$$\leftarrow \;v_{i}$;22:
$\mathrm{Set}\;w_{t}=$ 0;23:
**If** $V_{U}$ UNOCCUPIED **then**24:

$\vec{V_{U}V_{c}}$$\;\mathrm{represent}\;\mathrm{the}\;\mathrm{extended}\;\mathrm{branches}\;\mathrm{of}\;P_{STC}$$,\;\mathrm{with}\;V_{U}$ as parent vertex;25:

**If** $V_{U}\notin P_{opt\text{-}list}$26:


$\mathrm{Add}\;V_{U}$$\;\mathrm{to}\;\mathrm{the}\;\mathrm{end}\;\mathrm{of}\;\mathrm{the}\;P_{opt\text{-}list}$;27:


$w_{t}=w_{t}+1$;28:


**end**
29:
**Else if** $V_{L}$ UNOCCUPIED **then**30:

$\vec{V_{L}V_{c}}$$\;\mathrm{represent}\;\mathrm{the}\;\mathrm{extended}\;\mathrm{branches}\;\mathrm{of}\;P_{STC}$$,\;\mathrm{with}\;V_{L}$ as parent vertex;31:

**If** $V_{L}\notin P_{opt\text{-}list}$32:


$\mathrm{Add}\;V_{L}$$\;\mathrm{to}\;\mathrm{the}\;\mathrm{end}\;\mathrm{of}\;\mathrm{the}\;P_{opt\text{-}list}$;33:


$w_{t}=w_{t}+1$;34:


**end**
35:
**else if** $V_{D}$ UNOCCUPIED **then**36:

$\vec{V_{D}V_{c}}$$\;\mathrm{represent}\;\mathrm{the}\;\mathrm{extended}\;\mathrm{branches}\;\mathrm{of}\;P_{STC}$$,\;\mathrm{with}\;V_{D}$ as parent vertex;37:

**If** $V_{D}\notin P_{opt\text{-}list}$38:


$\mathrm{Add}\;V_{D}$$\;\mathrm{to}\;\mathrm{the}\;\mathrm{end}\;\mathrm{of}\;\mathrm{the}\;P_{opt\text{-}list}$;39:


$w_{t}=w_{t}+1$;40:


**end**
41:
**else if** $V_{R}$ UNOCCUPIED **then**42:

$\vec{V_{R}V_{c}}$$\;\mathrm{represent}\;\mathrm{the}\;\mathrm{extended}\;\mathrm{branches}\;\mathrm{of}\;P_{STC}$$,\;\mathrm{with}\;V_{R}$ as parent vertex;43:

**If** $V_{R}\notin P_{opt\text{-}list}$44:


$\mathrm{Add}\;V_{D}$$\;\mathrm{to}\;\mathrm{the}\;\mathrm{end}\;\mathrm{of}\;\mathrm{the}\;P_{opt\text{-}list}$;45:


$w_{t}=w_{t}+1$;46:


**end**
47:

**else**
48:

$V_{c}$ is the deadlock point;49:

**end if**
50:
$i=w_{t}$;51:
$\mathrm{Update}\;P_{STC}$;52:
$\mathrm{Update}\;P_{opt\text{-}list}$; 53:
$\mathrm{Remove}\;\mathrm{the}\;\mathrm{points}\;\mathrm{of}\;P_{STC}$$\;\mathrm{from}\;P_{opt\text{-}list}$;54:
$\mathrm{Update}\;\mathrm{the}\;\mathrm{point}\;\mathrm{with}\;\mathrm{the}\;\mathrm{end}\;\mathrm{of}\;\mathrm{the}\;P_{opt\text{-}list}$$\;\mathrm{to}\;\mathrm{the}\;V_{c}$;55:
**End**

56:

$\mathrm{Update}\;P_{STC};$


57:**Return** $P_{STC}$


### 3.4. Balanced Cut of the Explicit Coverage Path for STC

**Figure 7:**
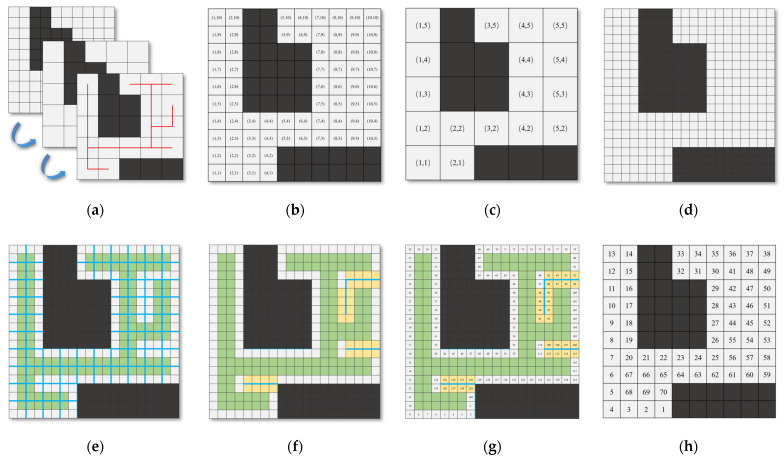
Flowchart of explicit STC coverage path. The black background is the real obstacle, and the gray-white background is the free area. In addition, the red line represents the proposed STC solution, the blue line represents the first-type virtual obstacle, the green background represents the second-type virtual obstacle, and the yellow background indicates that it is necessary to judge whether the robot has avoided the first-type virtual obstacle in this area.: (**a**) flowchart for obtaining the STC solution based on the original grid map; (**b**) original grid coordinates at the corresponding positions in $M_{R}$; (**c**) coordinates at the corresponding positions in the $M_{STC}$; (**d**) illustration of coordinates at corresponding positions in $M_{v}$; (**e**) illustration of the unprocessed $M_{v}$ with first-type obstacle and second-type obstacle; (**f**) illustration of $M_{v}$ after processing with Algorithm 2; (**g**) reference path list $P_{cover}\;$ obtained from solving $M_{v}$; (**h**) path-list $P_{all}$ obtained by mapping to $M_{R}$.

To obtain an explicit STC path $P_{cover}$ for the scene $M_{R}$ and to balance segmentation the $P_{cover}$ to $Solution_{cpp}$= {$P_{1}$, $P_{2}$, $P_{3}$…} for every robot in the system, this study first uses Algorithm 1 to obtain the STC solution on the corresponding map $M_{STC}$ with dimensions. Its overall process flow is illustrated in Figure 7.

The method proposed in this study initializes a grid-map, $M_{v}$. Let the size of $M_{R}$ be m × n, then the size of $M_{STC}$ was m/2 × n/2. The corresponding size of $M_{v}$ is 2m × 2n, where the obstacles in $M_{R}$ are also mapped as obstacles in $M_{v}$. We can derive the quantification formulas for the corresponding positions between each matrix through simple scaling of the coordinate system. Additionally, we define the edges of all cells in $M_{R}$ as first-type virtual obstacles mapped to $M_{v}$. Furthermore, all STC-generated branches are defined as second-type virtual obstacles. When an edge of a first-type obstacle corresponds to $M_{R}$, it intersects with a branch corresponding to a second-type obstacle to remove the first-type obstacle. A loop can be obtained, as illustrated in Figure 7.

The first-type virtual obstacle is equivalent to the grid being occupied, and the second-type virtual obstacle needs to be maintained in a hash table. Each time a new path point is generated, it is determined whether the line segment corresponding to the path intersects with the virtual obstacle in the hash table.

**Algorithm 2** Balanced cut of the explicit coverage path**Input:** $\mathrm{STC}\;\mathrm{solution}:\;P_{STC}$ **Output:** $\mathrm{Coverage}\;\mathrm{path}:\;Solution_{cpp}$$=\{P_{1}$$,\;P_{2}$$,\;P_{3}$…}$S_{r}\leftarrow M_{R}$(UNOCCUPIED); $\mathrm{Initialize}\;\mathrm{the}\;\;C_{initial}$$\;\mathrm{by}\;M_{R}$;//Corresponding to the original grid map$\mathrm{Initialize}\;\mathrm{the}\;\;C_{STC}$$\;\mathrm{as}\;M_{STC}$;//Corresponding to the STC sulotion$\mathrm{Initialize}\;\mathrm{the}\;\;C_{virtual}$$\;\mathrm{as}\;M_{v}$;//Matrix containing virtual obstacles$M_{c}\leftarrow \left\{V_{L},\;V_{U},\;V_{R},\;V_{D}\right\}$; //Four points surrounding the current point1:$P_{STC\text{-}vector}\leftarrow \;P_{STC}$; //Vector matrix of each branch of the STC2:$P_{obs}\leftarrow \;C_{initial}$;

$//\mathrm{The}\;\mathrm{grid}\;\mathrm{edges}\;\mathrm{of}\;C_{initial}$


$\;\mathrm{are}\;\mathrm{obstacles}\;\mathrm{of}\;M_{v}$

3:$P_{obs\text{-}vector}\leftarrow \;P_{obs}$;

$//\mathrm{Vector}\;\mathrm{matrix}\;\mathrm{of}\;\mathrm{each}\;\mathrm{branch}\;\mathrm{of}\;\mathrm{the}\;P_{obs}$

4:$\mathrm{Sum}\;\mathrm{the}\;\mathrm{number}\;\mathrm{of}\;\mathrm{Vector}\;\mathrm{in}\;P_{obs\text{-}vector}$$\;\mathrm{as}\;I_{ov}$;5:$\mathrm{Sum}\;\mathrm{the}\;\mathrm{number}\;\mathrm{of}\;\mathrm{Vector}\;\mathrm{in}\;P_{STC\text{-}vector}$$\;\mathrm{as}\;I_{sv}$;6:$\mathrm{Sum}\;\mathrm{the}\;\mathrm{number}\;\mathrm{of}\;\mathrm{cell}\;\mathrm{in}\;M_{v}$$\;\mathrm{as}\;I_{mv}$;7:$S_{all}\leftarrow M_{v}$(UNOCCUPIED);
8:$\mathrm{Sum}\;\mathrm{the}\;\mathrm{number}\;\mathrm{of}\;\mathrm{cell}\;\mathrm{in}\;S_{all}$$\;\mathrm{as}\;I_{sa}$;9:**For** $i=1:I_{sv}$10:
$\mathrm{Extract}\;\mathrm{the}\;\mathrm{endpoints}\;\mathrm{and}\;\mathrm{line}\;\mathrm{segments}\;\mathrm{of}\;\mathrm{vector}\;P_{STC\text{-}vector}$;11:
**For** $j=1:I_{ov}$12:

**If** $P_{obs\text{-}vector}$$\;\mathrm{intersects}\;\mathrm{with}\;P_{STC\text{-}vector}$ at a point or line segment13:


**Remove** $P_{obs\text{-}vector}$**from** $P_{obs}$;14:


**End**
15:

**End**
16:
**End**
17:$\mathrm{Update}\;P_{obs}$;18:**For** $i=1:S_{all}$19:
**For** $j=1:I_{sv}$20:

**If** $v_{mv}$$\;\mathrm{intersects}\;\mathrm{with}\;P_{STC\text{-}vector}$ at a point or line segment21:


$M_{v}$$(\mathrm{OCCUPIED})\;\leftarrow v_{mv}$;22:


**End**
23:

**End**
24:
**End**
25:$\mathrm{Update}\;M_{v}$;26:$\mathrm{Initialize}\;a\;\mathrm{random}\;\mathrm{starting}\;\mathrm{point}\;v_{i}\;$$\mathrm{in}\;M_{v}$;27:$\mathrm{Initialize}\;a\;\mathrm{path}\;\mathrm{list}\;M_{v}$$\;\mathrm{as}\;P_{all}$;28:**While** $i\leftarrow I_{sa}$29:
$V_{c}$$\leftarrow \;v_{i}$;30:
$\mathrm{Set}\;w_{t}=$ 0;31:
**If** $V_{U}$$\;\mathrm{UNOCCUPIED}\;\mathrm{and}\;\mathrm{not}\;\mathrm{in}\;\mathrm{the}\;P_{all}$ **then**32:

**If** $\vec{V_{U}V_{c}}$$\;\mathrm{not}\;\mathrm{intersects}\;\mathrm{with}\;\mathrm{any}\;\mathrm{Vector}\;\mathrm{in}\;P_{obs\text{-}vector}$33:


$P_{all}\;\leftarrow V_{U}$;34:


$w_{t}=w_{t}+1$;35:


**end**
36:
**Else if** $V_{L}$$\;\mathrm{UNOCCUPIED}\;\mathrm{and}\;\mathrm{not}\;\mathrm{in}\;\mathrm{the}\;P_{all}$ **then**37:

**If** $\vec{V_{L}V_{c}}$$\;\mathrm{not}\;\mathrm{intersects}\;\mathrm{with}\;\mathrm{any}\;\mathrm{Vector}\;\mathrm{in}\;P_{obs\text{-}vector}$38:


$P_{all}\;\leftarrow V_{L}$;39:


$w_{t}=w_{t}+1$;40:


**end**
41:
**else if** $V_{D}$$\;\mathrm{UNOCCUPIED}\;\mathrm{and}\;\mathrm{not}\;\mathrm{in}\;\mathrm{the}\;P_{all}$ **then**42:

**If** $\vec{V_{D}V_{c}}$$\;\mathrm{not}\;\mathrm{intersects}\;\mathrm{with}\;\mathrm{any}\;\mathrm{Vector}\;\mathrm{in}\;P_{obs\text{-}vector}$43:


$P_{all}\;\leftarrow V_{D}$;44:


$w_{t}=w_{t}+1$;45:


**end**
46:
**else if** $V_{R}$$\;\mathrm{UNOCCUPIED}\;\mathrm{and}\;\mathrm{not}\;\mathrm{in}\;\mathrm{the}\;P_{all}$ **then**47:

**If** $\vec{V_{R}V_{c}}$$\;\mathrm{not}\;\mathrm{intersects}\;\mathrm{with}\;\mathrm{any}\;\mathrm{Vector}\;\mathrm{in}\;P_{obs\text{-}vector}$48:


$P_{all}\;\leftarrow V_{R}$;49:


$w_{t}=w_{t}+1$;50:


**end**
51:

**else**
52:

$V_{c}$ is the end point of the path;53:

**end if**
54:
$i=w_{t}$;55:
$\mathrm{Update}\;P_{all}$;56:
**End**

57:$\mathrm{Sum}\;\mathrm{the}\;\mathrm{number}\;\mathrm{of}\;\mathrm{cell}\;\mathrm{in}\;P_{all}$$\;\mathrm{as}\;I_{pa}$;
58:$\mathrm{Sum}\;\mathrm{the}\;\mathrm{number}\;\mathrm{of}\;\mathrm{cell}\;\mathrm{in}\;M_{R}$$\;\mathrm{as}\;I_{mr}$;
59:$\mathrm{Initialize}\;a\;\mathrm{path}\;\mathrm{list}\;M_{R}$$\;\mathrm{as}\;P_{cover}$;
60:$\mathrm{Initialize}\;a\;\mathrm{starting}\;\mathrm{point}\;V_{i}\;$$\mathrm{in}\;M_{R}$;
61:**For** $i=1:I_{mr}$
62:
**For** $j=1:I_{pa}$
63:

**If** $\mathrm{The}\;\mathrm{cells}\;\mathrm{in}\;P_{all}$$\;\mathrm{correspond}\;\mathrm{to}\;\mathrm{the}\;\mathrm{cells}\;\mathrm{in}\;M_{R}$ at matching positions
64:


$P_{cover}\;\leftarrow V_{i}$;
65:


**End**

66:

**End**

67:
**End**

68:$\mathrm{Remove}\;\mathrm{duplicate}\;\mathrm{points}\;\mathrm{from}\;P_{cover}$;
69:$\mathrm{Update}\;P_{cover}$;
70:

$\mathrm{Divide}\;\mathrm{the}\;\mathrm{continuous}\;\mathrm{path}\;\mathrm{into}\;R\;\mathrm{equal}\;\mathrm{parts},\;\mathrm{as}\;P_{1}$


$,\;P_{2}$


$,\;P_{3}\cdots ;$


71:$\mathrm{Update}\;Solution_{cpp}$$=\{P_{1}$$,\;P_{2}$$,\;P_{3}$…};
72:**Return** $Solution_{cpp}$$=\{P_{1}$$,\;P_{2}$$,\;P_{3}$…}


Additionally, this loop forms a Hamiltonian circuit, allowing coverage of the area starting from any point on the path and ensuring that the endpoint is adjacent to the starting point. This property is particularly advantageous for disinfection and cleaning robots operating in a cyclic mode, which is one of the main reasons why many researchers use the STC method to address such issues [38,39,40,41,42,43].

### 3.5. ROS Based Framework for MCPP

**Figure 8:**
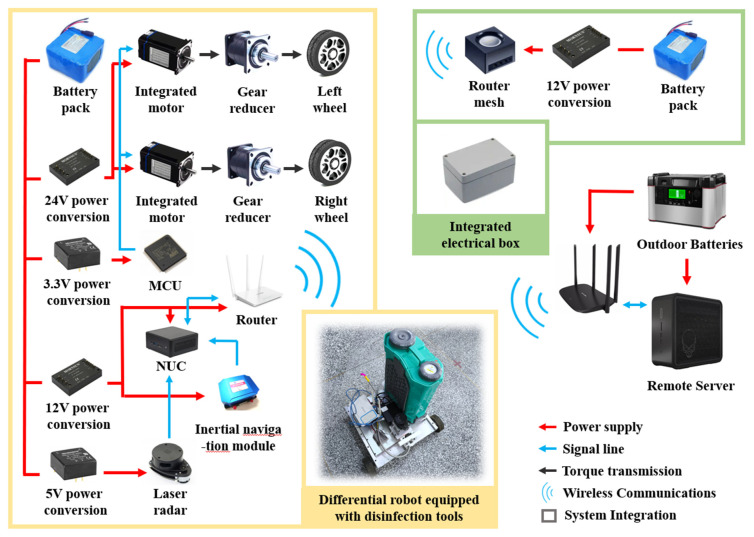
Differential robot platform for experimental validation.

This study investigated large-scale scenarios for industrial cleaning robots compatible with both indoor and outdoor environments, and found that few products fully implement kinematic constraints. Therefore, a differential-drive robot was designed and built, as shown in Figure 8. It achieves differential driving through two sets of motors, when the component model is ZLIS57C-10 Φ8 (The manufacturer is Shenzhen Zhongling Technology Co., Ltd., located in Shenzhen, Guangdong, China) and 57CLF gearboxes (The manufacturer is Beijing Times 4D Technology Co., Ltd., located in Beijing, China), based on the CANopen protocol. Communication with the motors is handled by the lower-level system, which integrates an STM32F407 chip (STMicroelectronics) and a CTM1051 module (The manufacturer is Zhiyuan Electronics Co., Ltd., located in Guangzhou, Guangdong, China). The upper-level system, consisting of modules like NUC (Intel, Ltd., Santa Clara, CA, USA) and Tenda routers (The manufacturer is Shenzhen Jixiang Tengda Technology Co., Ltd., located in Shenzhen, Guangdong, China), handles the transmission of kinematic commands to the lower system, as well as the collection of sensor information and the deployment of localization algorithms.

By publishing global path sequence points in real-time within the network via a TOPIC [44], the robot achieves navigation functionality. Cubic spline interpolation is applied to the global path reference points, and the Dynamic Window Approach (DWA) is used to track the smoothed path after interpolation.

**Figure 9:**
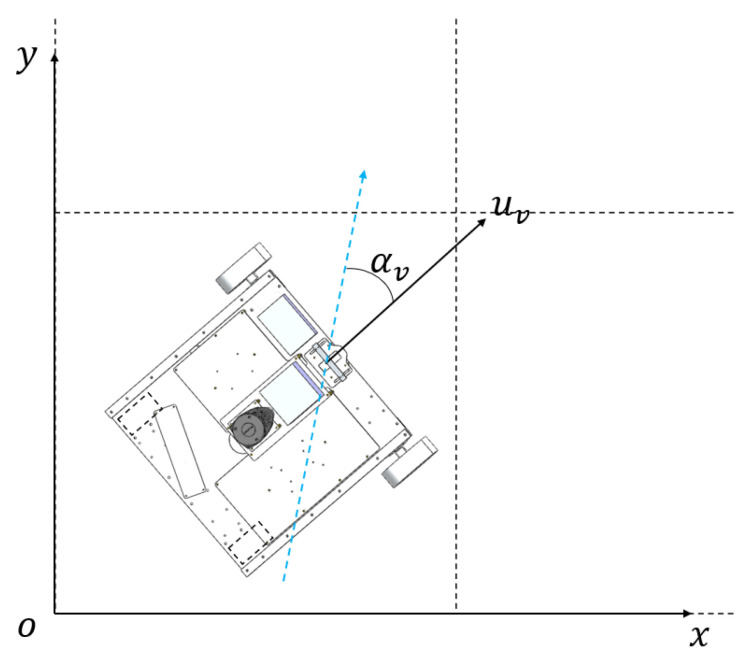
Kinematic model of the robot used to validate the method. The black arrow indicates the direction of the robot’s velocity, and the blue line indicates the target posture that needs to be achieved after receiving the deflection command.

The robot kinematic interface corresponding to the smooth path deployed on the robot is speed parameter and angular velocity parameter, where the speed is achieved by the rotation of the two driving wheels, and the angular velocity is achieved by the two driving wheels rotating in opposite directions. The kinematic diagram of the robot is shown in Figure 9.

To implement this framework, we preprocess the constructed map and use Octave to obtain the STC solution, generating explicit discrete global path points. These paths are then published to the differential-drive robot via the ROS Topic functions integrated in Simulink. The differential-drive robot is equipped with a 2D LIDAR and an inertial navigation module, with a cartographer deployed for localization.

Using the robot’s coordinates and the upcoming two consecutive discrete global path points, we apply cubic spline interpolation and compute the second derivative along the tangent direction as input for the DWA. When the Manhattan distance to the next global path point $e_{1}$ is less than $e_{h1}$, the next global path point is set as the target. If the current point’s error $e_{2}$ exceeds $e_{h2}$, the smooth local path is updated until all path points have been traversed. The values $e_{1}$ and $e_{2}$ are defined by Equations (9) and (10).

(9) $$e_{1}=\left|\left(x_{global}-x_{v}\right)\left|+\right|\left(y_{global}-y_{v}\right)\right|$$

(10) $$e_{2}=\max\left\{\left|\left(x_{global}-x_{v}\right)\left|,\right|\left(y_{global}-y_{v}\right)\right|\right\}$$

When the endpoint is the target point, and only two points remain between the target and the robot’s current location, the previous global path point is used as a supplementary point if the local path needs updating. Based on engineering experience, in this study, we set $e_{h1}$ = 0.25 m and $e_{h2}\;$= 0.10 m.

## 4. Experiment and Discussion

To verify the effectiveness of the method, this study conducted 210 simulation tests and performed experimental testing on a real robot.

Firstly, we set the lower boundary of single-robot operations to the standard size of a classic industrial cleaning robot, with the upper boundary based on the size of Beijing Universal Studios [45]. For different scene sizes, we generated 10 random maps per size based on a random seed, resulting in a total of 180 simulation tests to demonstrate the effectiveness of this study in social environments. For each environment size, a single case was selected to illustrate the effectiveness of the STC solution.

Under different environment sizes, obstacles are randomly generated and arranged in them, and then the total number of grids that need to be covered $S_{sum}$ can be obtained. $T_{stc}$ represents the solution time of the method in the literature, $T_{our}$ represents the solution time of the proposed method, and the results of this study are obtained through secondary development, that is, the percentage of calculation time reduction ($T_{r}$). The above test results are shown in Table 2. In order to save space, we provide 40 sets of scene data to prove the superiority of our method.

**Figure 10:**
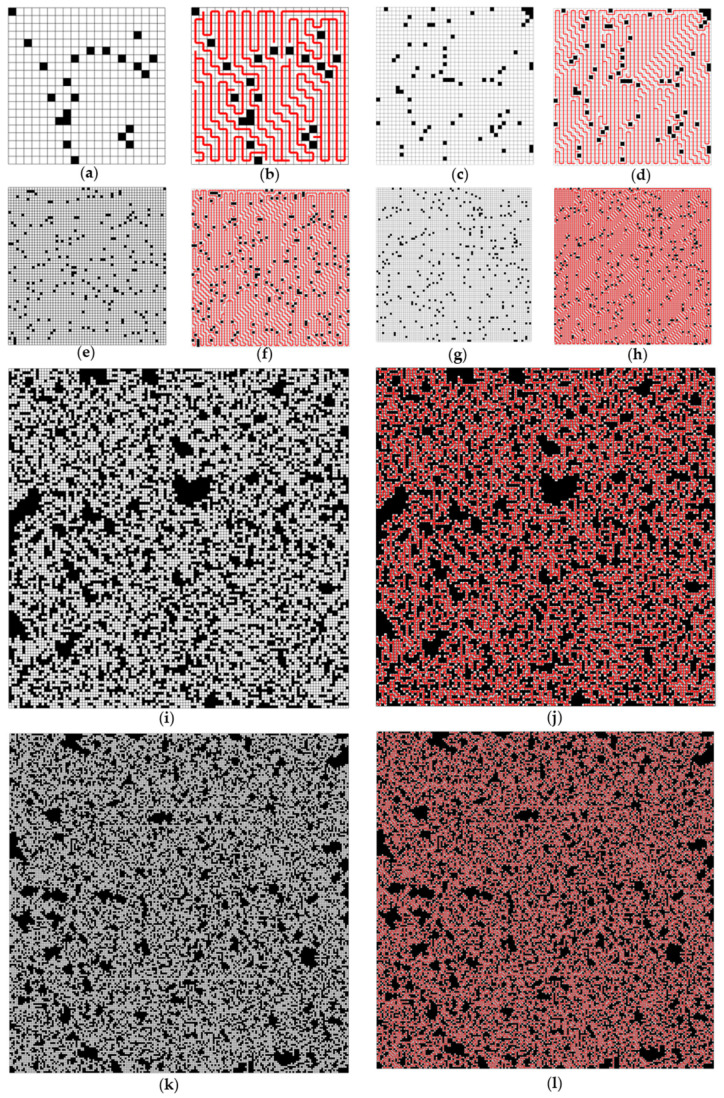
Environments and solution cases in six conditions, The black part in the figure represents the obstacles, and the red line represents the obtained STC solution: (**a**) size 40 × 40; (**b**) size 40 × 40 solution; (**c**) size 80 × 80; (**d**) size 80 × 80 solution; (**e**) size 120 × 120; (**f**) size 120 × 120 solution; (**g**) size 160 × 160; (**h**) size 160 × 160 solution; (**i**) size 250 × 250; (**j**) size 250 × 250 solution; (**k**) size 400 × 400; (**l**) size 400 × 400 solution.

The test results indicate that the proposed method can effectively reduce computation time, achieving a reduction of up to 82.47% in favorable cases. The underlying principles of our method’s superiority have been discussed in detail in Section 3.3. Our method was applied to six scenarios with sizes of 40 × 40, 80 × 80, 120 × 120, 160 × 160, 250 × 250, and 400 × 400, along with the corresponding solution cases, as shown in Figure 10.

Mainstream methods that consider computational complexity include RW (Random Walk), DFS (Depth First Search) [46], and DARP [47]. Additionally, the BP [48,49] method introduces new TSP issues due to region partitioning and does not address path conflicts between multiple robots. Other offline methods, such as MSTC and MIP, can obtain optimal solutions, but their high computational complexity makes them challenging to apply in large-scale scenarios and, thus, they were not included in the comparisons in this study.

We generated 10 random obstacle-containing scenarios of size 80 × 80 and conducted 30 simulation tests using the methods RW, DARP (both effective in obstacle environments), and our proposed method. We compared the results based on three metrics: coverage rate, secondary coverage rate, and system operation time. The results are shown in Table 3.

The results show that our method shows significant advantages in terms of secondary coverage and operation time. RW shows considerable advantages in unknown scenarios due to its high frequency of solution updates and ability to be deployed in unknown environments. Its frequent updates to local paths help mitigate path conflicts during multi-robot collaboration by maintaining a priority queue for robots in the cloud. However, in large known scenes, the inherent stochasticity of RW can lead to excessive secondary coverage, resulting in longer overall operation times.

The RW and DFS methods have higher quadratic coverage, which indirectly leads to longer total coverage paths. At the same time, the solution of the DARP method does not show obvious advantages in the comparison of multiple groups of random environments.

**Figure 11:**
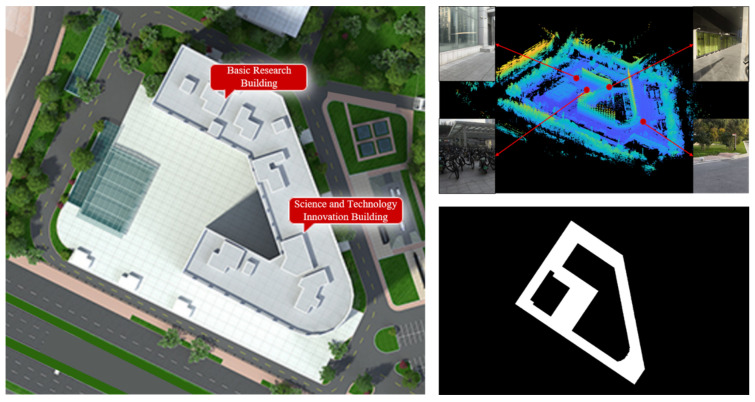
Instance scenario environment construction and grid map conversion. The figure in the lower right corner is a rasterized map of the area to be covered after processing, where the white part represents the area to be covered and the black part represents the obstacles.

Through the aforementioned simulation tests, the robots deployed in their operational areas for scenarios not exceeding the dimensions of 80 × 80 exhibit a re-planning time of less than 3 s. This means that when the robots navigate between two adjacent global path points that are 1 m apart at a typical operational speed of 0.35 m/s, they can successfully complete the re-planning process when new obstacles appear. This capability meets the demands of practical applications, rendering the DARP method unable to demonstrate a clear advantage in the issues discussed in this study. Finally, we constructed a scene model near the HIT-BIT building and transformed it into a two-dimensional map through processing, as shown in Figure 11.

**Figure 12:**
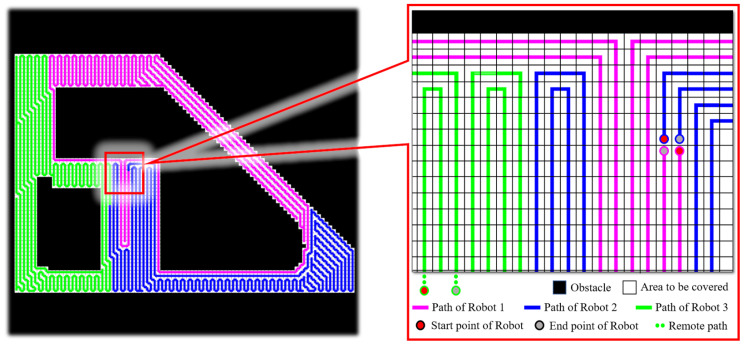
Three-robot coverage path generation.

Based on the framework proposed in Section 3.5, the solved path was published to the differential robot for hardware validation, and the results are shown in Figure 12.

After processing the three-dimensional point cloud, a grid map with a resolution of 1 m^2^ was obtained. The total number of grids that need to be covered in this map is 9500. After being solved by the proposed method, the number of grids assigned to each robot was 3166, 3166, and 3168, respectively, and the solution time was 1.6 s. Based on the robot referenced in this study, when covering the operation at a speed of 0.35 m/s, the passing time between two grids was 2.85 s. If a person or vehicle occupies the next grid in the robot’s global reference path at the next moment, the system can achieve re-planning capability.

## 5. Conclusions

This study first innovatively introduced the concept of superior backtracking points and explained the principle of reducing the number of backtracking times during the backtracking process. Then, based on the original SCAN-STC algorithm, by additionally maintaining two designed data lists, the algorithm starts backtracking from the superior backtracking point in the process of solving the problem, which reduces the algorithm solution time and ultimately achieves the purpose of reducing computational complexity at the expense of space complexity.

And through secondary encoding, the STC solution is explicitly represented as a continuous path, and finally the cut is balanced. Finally, a framework is provided to illustrate the method’s input requirements and kinematic requirements for the deployment platform.

This study also indirectly illustrates the generalization ability of the method in reducing computational complexity by proving the necessity of backtracking. The effectiveness of the method was proved by simulation tests in a series of random environments with other recent methods in the literature, and the calculation time was reduced by 82.47% in the best case.

In 10 random environments containing obstacles, it was compared with the RW and DFS methods, with path planning as the main module, and the DARP method with region segmentation as the main module. The results show that in this type of environment, the RW and DFS methods have high real-time performance, but high quadratic coverage. The solution of the DARP method does not show obvious advantages in the comparison of multiple groups of random environments.

Our method achieves a high update frequency while ensuring low quadratic coverage, improving the robot’s path re-planning ability when performing coverage tasks in social environments.

With the support of the new project, we will optimize the bandwidth of data synchronization between robots to achieve the ability of system information sharing in hardware. At the same time, by optimizing the robot dynamics, we will further improve the algorithm’s ability to resist interference from the external environment when facing outdoor unevenness, and provide better protection for the online solution of MCPP problems in social environments.

## Figures and Tables

**Table 1:** Parameters and settings for modeling.

Parameters	Settings
*r*	Set of robots’ labels for CPP task, indexed by r
*R*	Set of robot number for CPP task
$I_{h}$	The number of iterations in the algorithm for STC
$S_{1}$	The area that robot number 1 needs to cover
$S_{all}$	The area that all robots in the system need to cover
$M_{R}$	The grid-map corresponding to the CPP problem
$v_{r}$	Points on the coverage path of robot numbered r
$w_{r}$	Total workload of robot numbered r
$S_{t}$	Maximum threshold of the intersection of the covered areas
$G_{opt}$	The difference between obtained solution and optimal solution
C	$\mathrm{Represents}\;\mathrm{the}\;\mathrm{matrix},\;\mathrm{where}\;C_{1}$ denotes matrix numbered as 1
$l_{evn}$	The width of the environment under study
$h_{evn}$	The length of the environment under study
$N_{obs}$	The number of cells occupied by obstacles in grid map
$P_{STC}$	The real-time STC solution queue
$P_{opt\text{-}list}$	The real-time updated superior backtracking point list
$P_{boundary\text{-}next}$	The obstacle-adjacent point list
$M_{c}$	The cells list which around the vertex
$v_{c}$	The current point used in the loop instruction of the algorithm
$v_{next}$	The Adjacent point used in the instructions of the algorithm
$V_{c}$	One end of the vector or line segment
$M_{v}$	Virtual grid map required for balance cut Algorithm
$M_{stc}$	The STC solution map for Algorithm
$C_{initial}$	Matrix corresponding to the original grid map
$C_{stc}$	Matrix corresponding to the STC grid map
$C_{virtual}$	$\mathrm{Matrix}\;\mathrm{corresponding}\;\mathrm{to}\;\mathrm{the}\;\mathrm{grid}\;\mathrm{map}\;M_{v}$
$P_{obs}$	$\mathrm{The}\;\mathrm{grid}\;\mathrm{edges}\;\mathrm{of}\;C_{initial}$
$P_{STC\text{-}vector}$	Vector matrix of each branch of the STC solution
$P_{obs\text{-}vector}$	$\mathrm{Vector}\;\mathrm{matrix}\;\mathrm{of}\;\mathrm{each}\;\mathrm{branch}\;\mathrm{of}\;P_{obs}$
$I_{ov}$	$\mathrm{The}\;\mathrm{number}\;\mathrm{of}\;\mathrm{Vector}\;\mathrm{in}\;P_{obs\text{-}vector}$
$I_{sv}$	$\mathrm{The}\;\mathrm{number}\;\mathrm{of}\;\mathrm{Vector}\;\mathrm{in}\;P_{STC\text{-}vector}$
$I_{mv}$	$\mathrm{The}\;\mathrm{number}\;\mathrm{of}\;\mathrm{cells}\;\mathrm{in}\;M_{v}$
$I_{sa}$	$\mathrm{The}\;\mathrm{number}\;\mathrm{of}\;\mathrm{cells}\;\mathrm{in}\;S_{all}$
$P_{all}$	$\;\mathrm{Feasible}\;\mathrm{solution}\;\mathrm{for}\;a\;\mathrm{complete}\;\mathrm{coverage}\;\mathrm{path}\;\mathrm{in}\;M_{v}$
$I_{pa}$	$\mathrm{The}\;\mathrm{number}\;\mathrm{of}\;\mathrm{cells}\;\mathrm{in}\;P_{all}$
$I_{mr}$	$\mathrm{The}\;\mathrm{number}\;\mathrm{of}\;\mathrm{cells}\;\mathrm{in}\;M_{R}$
$V_{i}$	The points with STC solution explicitization path
$P_{r}$	The coverage path assigned to the robot, indexed by r
$e_{1}$	Global Navigation Path Error Value
$e_{2}$	Local Planning Path Error Value
$e_{h1}$	Permissible Error for Global Navigation Planning
$e_{h2}$	Permissible Error for Local Navigation Planning
$x_{global}$	Global Target Point coordinate value in the X Direction
$y_{global}$	Global Target Point coordinate value in the Y Direction
$x_{v}$	X Coordinate Value of the Robot in Global Coordinates
$y_{v}$	Y Coordinate Value of the Robot in Global Coordinates
$N_{sum}$	Total grid number to be covered for comparison
$T_{stc}$	Solution time of the relatively advanced STC method
$T_{our}$	Solution time of our STC method
$T_{r}$	The solution time reduction percentage

**Table 2:** Reduction in computational time by ours in the selected 40 random scenarios.

Size of Case	$S_{sum}$	$T_{stc}$ (s)	$T_{our}$ (s)	$T_{r}$ (%)	Size of Case	$S_{sum}$	$T_{stc}$ (s)	$T_{our}$ (s)	$T_{r}$ (%)
40 × 40	1500	1.1186	0.2216	80.19	200 × 200	23,764	772.9788	209.1804	72.94
	1508	1.1669	0.2372	79.67		24,176	597.5242	241.9513	59.51
	1532	1.3108	0.3088	76.44		23,848	504.8885	214.2867	57.56
	1536	1.2728	0.2697	78.81		22,628	646.2358	239.2903	62.97
	1524	1.3639	0.2390	82.47		24,828	759.1654	203.2268	73.23
80 × 80	6056	8.2671	2.2079	73.29	250 × 250	38,972	2020.1102	680.0454	66.34
	6120	5.3840	1.5339	71.51		37,360	1570.5011	656.2255	58.18
	6104	6.3608	2.0117	68.37		37,180	1691.3564	746.5494	55.86
	6104	5.6826	1.7120	69.87		38,448	1433.5215	598.2217	58.27
	6104	6.5864	2.2201	66.29		39,012	1717.2946	741.4400	56.84
120 × 120	13,632	29.6055	12.7036	57.09	350 × 350	75,428	3103.9135	1623.3156	47.69
	13,712	20.1042	7.0489	64.94		72,072	4022.1956	1693.3251	57.91
	13,736	29.3870	12.2862	58.19		76,792	3552.5219	1680.9143	52.70
	13,684	22.0293	8.8440	59.85		76,156	4498.8956	1708.1125	62.03
	13,668	25.0810	8.9820	64.18		73,840	3049.8012	1417.4019	53.55
160 × 160	24,252	87.2286	34.9469	59.93	400 × 400	97,760	15,051.1254	4435.7156	70.53
	24,340	86.7675	32.2355	62.84		96,436	14,480.5340	4740.9543	67.26
	24,440	77.7163	33.8955	56.38		97,724	14,406.0011	4376.3659	69.62
	24,276	74.8750	28.2694	59.84		97,576	9805.2450	4612.1524	52.96
	24,404	75.9683	34.1139	55.09		98,168	9842.7152	4616.5214	53.11

**Table 3:** Comparative simulation testing of different algorithms.

Case Label	Quantitative Comparison Items	RW	DFS	DARP	Ours
1	Coverage rate	90.62%	93.98%	100%	100%
	Secondary coverage	315.21%	17.18%	0%	0%
	Total path length	6283	1774	1514	1514
	Update frequency	8.33 Hz	5.52 Hz	0.03 Hz	0.40 Hz
2	Coverage rate	92.49%	92.45%	100%	100%
	Secondary coverage	152.21%	21.51%	0%	0%
	Total path length	0.09 s	1859	1530	1530
	Update frequency	11.11 Hz	4.21 Hz	0.05 Hz	0.53 Hz
3	Coverage rate	91.11%	91.12%	100%	100%
	Secondary coverage	255.21%	32.48%	0%	0%
	Total path length	5630	2093	1586	1586
	Update frequency	11.11 Hz	5.15 Hz	0.19 Hz	0.42 Hz
4	Coverage rate	93.10%	94.49%	100%	100%
	Secondary coverage	145.21%	42.12%	0%	0%
	Total path length	3848	2230	1571	1571
	Update frequency	10.00 Hz	2.18 Hz	0.14 Hz	0.52 Hz
5	Coverage rate	90.98%	93.01%	100%	100%
	Secondary coverage	385.21%	35.24%	0%	0%
	Total path length	7478	2081	1542	1542
	Update frequency	9.09 Hz	3.15 Hz	0.03 Hz	0.51 Hz
6	Coverage rate	88.34%	90.11%	100%	100%
	Secondary coverage	485.21%	25.69%	0%	0%
	Total path length	8769	1873	1499	1499
	Update frequency	11.11 Hz	6.01 Hz	0.05 Hz	0.52 Hz
7	Coverage rate	91.28%	92.52%	100%	100%
	Secondary coverage	354.21%	46.11%	0%	0%
	Total path length	6819	2177	1502	1502
	Update frequency	10.00 Hz	3.11 Hz	0.05 Hz	0.49 Hz
8	Coverage rate	89.05%	88.98%	100%	100%
	Secondary coverage	264.21%	19.77%	0%	0%
	Total path length	5179	1693	1423	1423
	Update frequency	7.69 Hz	2.45 Hz	0.05 Hz	0.41 Hz
9	Coverage rate	93.31%	95.09%	100%	100%
	Secondary coverage	157.21%	45.29%	0%	0%
	Total path length	3957	2233	1540	1540
	Update frequency	7.14 Hz	2.00 Hz	0.16 Hz	0.81 Hz
10	Coverage rate	91.22%	91.74%	100%	100%
	Secondary coverage	246.21%	46.15%	0%	0%
	Total path length	4927	2079	1424	1424
	Update frequency	12.5 Hz	4.98 Hz	0.09 Hz	0.79 Hz

## Data Availability

Data sharing is not applicable.

## References

[1] Chen Y., Luo Y., Ozkan Yerebakan M., Hu B. (2022). Human Acceptance of the Cleaning Robot in Grocery Environments During the COVID-19 Pandemic. Proceedings of the Human Factors and Ergonomics Society Annual Meeting.

[2] Mavrogiannis C., Baldini F., Wang A., Zhao D., Trautman P., Steinfeld A., Oh J. (2023). Core challenges of social robot navigation: A survey. ACM Trans. Hum.-Robot. Interact..

[3] Aldinhas Ferreira M.I., Sequeira J.S., Aldinhas Ferreira M., Silva Sequeira J., Tokhi M., E. Kadar E., Virk G. (2017). Robots in Ageing Societies. A World with Robots. Intelligent Systems, Control and Automation: Science and Engineering.

[4] Garcia-Haro J.M., Oña E.D., Hernandez-Vicen J., Martinez S., Balaguer C. (2020). Service robots in catering applications: A review and future challenges. Electronics.

[5] Shin H., Kang J. (2020). Reducing perceived health risk to attract hotel customers in the COVID-19 pandemic era: Focused on technology innovation for social distancing and cleanliness. Int. J. Hosp. Manag..

[6] Dogru S., Marques L. (2022). ECO-CPP: Energy constrained online coverage path planning. Robot. Auton. Syst..

[7] Bradner K.M. (2020). Path Planning for Variable Scrutiny Multi-Robot Coverage. Master’s Thesis.

[8] Tang J., Ma H. (2023). Mixed Integer Programming for Time-Optimal Multi-Robot Coverage Path Planning with Heuristics. IEEE Robot. Autom. Lett..

[9] Lin Q. (2020). Design Characteristics of Culturally-Themed Luxury Hotel Lobbies in Las Vegas: Perceptual, Sensorial, and Emotional Impacts of Fantasy Environments. Master’s Thesis.

[10] Liu L., Wang X., Yang X., Liu H., Li J., Wang P. (2023). Path planning techniques for mobile robots: Review and prospect. Expert Syst. Appl..

[11] Wang N., Yang X., Wang T., Xiao J., Zhang M., Wang H., Li H. (2023). Collaborative path planning and task allocation for multiple agricultural machines. Comput. Electron. Agric..

[12] Abdulsaheb J.A., Kadhim D.J. (2023). Classical and heuristic approaches for mobile robot path planning: A survey. Robotics.

[13] Tonola C., Faroni M., Pedrocchi N., Beschi M. (2021). Anytime informed path re-planning and optimization for human-robot collaboration. Proceedings of the 2021 30th IEEE International Conference on Robot & Human Interactive Communication (RO-MAN).

[14] Pedergnana A., Calandra I., Bob K., Gneisinger W., Paixao E., Schunk L., Hildebrandt A., Marreiros J. (2020). Evaluating the microscopic effect of brushing stone tools as a cleaning procedure. Quat. Int..

[15] Karwowski J., Szynkiewicz W. (2023). Quantitative metrics for benchmarking human-aware robot navigation. IEEE Access.

[16] Kumar K., Kumar N. (2023). Region coverage-aware path planning for unmanned aerial vehicles: A systematic review. Phys. Commun..

[17] Glorieux E., Franciosa P., Ceglarek D. (2020). Coverage path planning with targetted viewpoint sampling for robotic free-form surface inspection. Robot. Comput. Integr. Manuf..

[18] Prassler E., Munich M.E., Pirjanian P., Kosuge K. (2016). Domestic robotics. Springer Handbook of Robotics.

[19] Gabriely Y., Rimon E. (2001). Spanning-tree based coverage of continuous areas by a mobile robot. Ann. Math. Artif. Intell..

[20] Viet H.H., Dang V.H., Laskar M.N., Chung T. (2013). BA*: An online complete coverage algorithm for cleaning robots. Appl. Intell..

[21] Acar E.U., Choset H. (2002). Sensor-based coverage of unknown environments: Incremental construction of morse decompositions. Int. J. Robot. Res..

[22] Liu H., Dong W., Zhang Z., Wang C., Li R., Gao Y. (2024). Optimization-based local planner for a nonholonomic autonomous mobile robot in semi-structured environments. Robot. Auton. Syst..

[23] Zhang B., Liu Y., Lu Q., Wang J. (2016). A path planning strategy for searching the most reliable path in uncertain environments. Int. J. Adv. Robot. Syst..

[24] Tolstaya E., Paulos J., Kumar V., Ribeiro A. (2021). Multi-robot coverage and exploration using spatial graph neural networks. Proceedings of the 2021 IEEE/RSJ International Conference on Intelligent Robots and Systems (IROS).

[25] Amorim D., Ventura R. (2015). A physics-based optimization approach for path planning on rough terrains. Proceedings of the 2015 12th International Conference on Informatics in Control, Automation and Robotics (ICINCO).

[26] Zhong Y., Shirinzadeh B., Yuan X. (2011). Optimal robot path planning with cellular neural network. Int. J. Intell. Mechatron. Robot. (IJIMR).

[27] Zhou Q., Gao S., Qu B., Gao X., Zhong Y. (2022). Crossover recombination-based global-best brain storm optimization algorithm for uav path planning. Proc. Rom. Acad. Ser. A-Math. Phys. Tech. Sci. Inf. Sci..

[28] Bahwini T., Zhong Y., Gu C. (2019). Path planning in the presence of soft tissue deformation. Int. J. Interact. Des. Manuf. (IJIDeM).

[29] Hills J., Zhong Y. (2014). Cellular neural network-based thermal modelling for real-time robotic path planning. Int. J. Agil. Syst. Manag. 20.

[30] Zhong Y., Shirinzadeh B., Tian Y. (2008). A new neural network for robot path planning. Proceedings of the2008 IEEE/ASME International Conference on Advanced Intelligent Mechatronics.

[31] Choset H., Pignon P. (1998). Coverage path planning: The boustrophedon cellular decomposition. Field and Service Robotics.

[32] Zhang B., Hong T., Xiong R., Chepinskiy S.A. (2021). A terrain segmentation method based on pyramid scene parsing-mobile network for outdoor robots. Int. J. Adv. Robot. Syst..

[33] Tang J., Sun C., Zhang X. (2021). MSTC∗: Multi-robot coverage path planning under physical constrain. Proceedings of the 2021 IEEE International Conference on Robotics and Automation (ICRA).

[34] Lu J., Zeng B., Tang J., Lam T.L. (2022). Tmstc*: A turn-minimizing algorithm for multi-robot coverage path planning. arXiv.

[35] Yang Y., He D., Mao H., Chen H., Wu H., Liu Z.W. (2023). RP-MSTC*: Multi-Agent Coverage Path Planning Algorithm Emphasizing Local Area Updates. Proceedings of the 2023 International Conference on Neuromorphic Computing (ICNC).

[36] Vandermeulen I., Groß R., Kolling A. (2019). Turn-minimizing multirobot coverage. Proceedings of the 2019 International Conference on Robotics and Automation (ICRA).

[37] Nair V.G., Guruprasad K.R. (2020). Geodesic-VPC: Spatial partitioning for multi-robot coverage problem. Int. J. Robot. Autom..

[38] Gabriely Y., Rimon E. (2002). Spiral-STC: An on-line coverage algorithm of grid environments by a mobile robot. Proceedings of the 2002 IEEE International Conference on Robotics and Automation (Cat. No. 02CH37292).

[39] Guruprasad K.R. X-stc: An extended spanning tree-based coverage algorithm for mobile robots. Proceedings of the 2019 4th International Conference on Advances in Robotics.

[40] Gao G.Q., Xin B. (2019). A-STC: Auction-based spanning tree coverage algorithm for motion planning of cooperative robots. Front. Inf. Technol. Electron. Eng..

[41] Tang J., Ma H. (2024). Large-Scale Multi-Robot Coverage Path Planning via Local Search. Proc. AAAI Conf. Artif. Intell..

[42] Gabriely Y., Rimon E. (2003). Competitive on-line coverage of grid environments by a mobile robot. Comput. Geom..

[43] Thiayagarajan K., Balaji C.G. (2012). Traversal algorithm for complete coverage. J. Comput. Sci..

[44] Hazem Z.B., Ince R., Dilibal S. Joint Control Implementation of 4-DOF Robotic Arm Using Robot Operating System. Proceedings of the 2022 International Conference on Theoretical and Applied Computer Science and Engineering (ICTASCE).

[45] https://en.wikipedia.org/wiki/Universal_Studios_Beijing.

[46] Tan C.S., Mohd-Mokhtar R., Arshad M.R. (2021). A comprehensive review of coverage path planning in robotics using classical and heuristic algorithms. IEEE Access.

[47] Kapoutsis A.C., Chatzichristofis S.A., Kosmatopoulos E.B. (2017). DARP: Divide Areas Algorithm for Optimal Multi-Robot Coverage Path Planning. J. Intell. Robot Syst..

[48] Guastella D.C., Cantelli L., Giammello G., Melita C.D., Spatino G., Muscato G. (2019). Complete coverage path planning for aerial vehicle flocks deployed in outdoor environments. Comput. Electr. Eng..

[49] Chen G., Shen Y., Zhang Y., Zhang W., Wang D., He B. (2021). 2D multi-area coverage path planning using L-SHADE in simulated ocean survey. Appl. Soft Comput..

